# Defining clavicle growth in infancy using chest radiographs

**DOI:** 10.3389/fped.2024.1433472

**Published:** 2024-10-28

**Authors:** Yvonne Hadamek, Paul-Christian Krueger, Hans-Joachim Mentzel, Matthias Waginger

**Affiliations:** Section of Pediatric Radiology, Department of Radiology, Jena University Hospital, Jena, Germany

**Keywords:** clavicle development, bone development, reference values, premature infants, children, thoracic radiography

## Abstract

**Background:**

Despite the critical role of the clavicle in understanding growth and development in early childhood, there remains a notable paucity of comprehensive studies investigating clavicle growth patterns during this crucial period. This hinders our ability to establish normative growth parameters during these early life stages. Our study sought to measure clavicle dimensions and subsequently construct growth curves spanning from preterm infants to toddlers up to the age of 6 years by measuring routine chest radiographs. Differences between both sides of the body and between the sexes should be analysed. This aimed to provide a nuanced understanding of clavicle growth dynamics and offering a foundation for the establishment of normative values in this understudied context. In this retrospective study, children aged 23 weeks of gestation to 6 years who underwent a chest radiography between January 2010 and June 2020 were enrolled. A total of 5.311 potential radiographs was screened. Clavicle length and width were measured in all radiographs using the Centricity™ Universal Viewer. Statistical analysis was performed using SPSS®.

**Results:**

1.340 images met the quality criteria to be included in our study. The growth curves of clavicle lengths and widths showed a steadily increasing trend with age. Inclusion of premature infants in the age group of one month resulted in a decrease in this age group. Significant differences between both sides of the body and between the sexes could be shown. Measurements of clavicle length and width, derived from routine chest radiographs, are highly accurate. This accuracy underscores the potential for utilizing thoracic radiographs as a reliable tool for assessing clavicle growth in clinical settings or even forensic analysts. The establishment of reference values derived from our measurements provides a basis for normative growth parameters.

## 1 Introduction

The clavicle stands as a pivotal bony structure in the human skeleton, connecting the upper limb to the axial skeleton. It serves as a cornerstone for biomechanical stability and mobility but also contributes to breathing. Deformities occur, for example, in clavicular pseudarthrosis, mandibuloacral dysplasia, cleidocranial dysplasia, or congenital anterior shoulder (1–7). Despite its anatomical significance and functional importance in facilitating a wide range of upper limb movements, the exact clavicle growth and development in early childhood remains undefined. The paucity of comprehensive studies addressing the specific and normative growth curves of the clavicle during these formative years creates a critical gap in our understanding of pediatric skeletal development. Leveraging routine chest radiographs, we aimed to measure clavicle dimensions and subsequently construct growth curves spanning from preterm infants to toddlers up to the age of 6 years. Furthermore, the potential influence of biological sex and laterality on clavicle development were conducted. This study is not only important to improve our understanding of skeletal maturation, but also has profound implications for clinical diagnosis, therapeutic interventions and forensic analysis. We did contribute valuable insights that will enrich the field of pediatric skeletal development and form the basis for further research.

## 2 Material and methods

Our study received ethical approval from the local ethics committee under the reference number 2021-2246. The chest radiographs used in our investigation were sourced from the Institute's digital radiography database, Radiology Information System (RIS) Lorenzo RadCentre, I-Solutions Health GmbH [57.0.1326.0]. Our focus centered on radiographs of patients born and x-rayed within the gestational age range of 23–40 weeks, as well as radiographs of neonates, infants, and young children up to 6 years old. All radiographs were acquired between January 2010 and June 2020. Patient identities were consistently pseudonymous, using the hospital information system's (HIS) patient identification number (ID). Inclusion criteria required radiographs to distinctly display both clavicles and exhibit symmetry in dimensions and appearance. Exclusions encompassed radiographs lacking clarity in displaying both clavicles, those of poor quality, or showing asymmetry due to body rotation. Individuals with metabolic disorders or fractures were also excluded. Consequently, a total of 1,340 chest radiographs met the specified criteria.

To establish normal growth curves based on a substantial number of radiographs, we categorized the subjects into age groups. as outlined in Table 1:

• Premature infants: less than 38 weeks of gestation
• Full-term infants: 38–42 weeks of gestation (up to 28 days of life)
• Infants: from the fourth week of life to the end of the first year
• Toddlers: from the second to the sixth year of life

**Table 1:** Data for each age group is presented in both absolute and relative terms, categorized by sex assigned at birth and overall.

Age group	*n*	*n* (boys)	*n* (girls)
23rd–26th week of gestation	51 (3.8%)	28 (3.8%)	23 (3.8%)
27th–30th week of gestation	73 (5.4%)	48 (6.5%)	25 (4.1%)
31st–34th week of gestation	58 (4.3%)	30 (4.1%)	28 (4.6%)
35th–37th week of gestation	45 (3.4%)	25 (3.4%)	20 (3.3%)
38th–40th week of gestation	106 (7.9%)	56 (7.6%)	50 (8.2%)
1 month	41 (3.1%)	23 (3.1%)	18 (3.0%)
2–4 months	42 (3.1%)	29 (4.0%)	13 (2.1%)
5–8 months	60 (4.5%)	37 (5.0%)	23 (3.8%)
9–12 months	43 (3.2%)	27 (3.7%)	16 (2.6%)
1 year	205 (15.3%)	115 (15.7%)	90 (14.8%)
2 years	188 (14.0%)	101 (13.8%)	87 (14.3%)
3 years	135 (10.1%)	68 (9.3%)	67 (11.0%)
4–6 years	293 (21.9%)	146 (19.9%)	147 (24.2%)
Total	**1,340** **(100%)**	**733** **(100%)**	**607** **(100%)**

The total number of images, along with the corresponding genders and their respective percentages, are highlighted in bold.

For premature infants, age corrections were not applied beyond the neonatal period of 28 days. Groups were defined so that each age group was completed, but the next group was not yet reached. (Further subdivisions within the groups aimed to capture more detailed growth patterns).

The accuracy of clavicle dimensioning in the radiographs was validated through a comparison with macerated bones. Manual measurements were initially conducted on the macerated bones, followed by x-ray imaging of clavicles using four common techniques for direct size comparison.

The radiographic techniques employed were as follows:

“Mobile x-ray unit; detector in incubator directly under the clavicle”
“Mobile x-ray unit; detector in incubator in slide-in module”
“Stationary x-ray unit; detector directly under the clavicle, with grid”
“Stationary x-ray unit; detector directly under clavicle; without grid”

**Figure 1:**
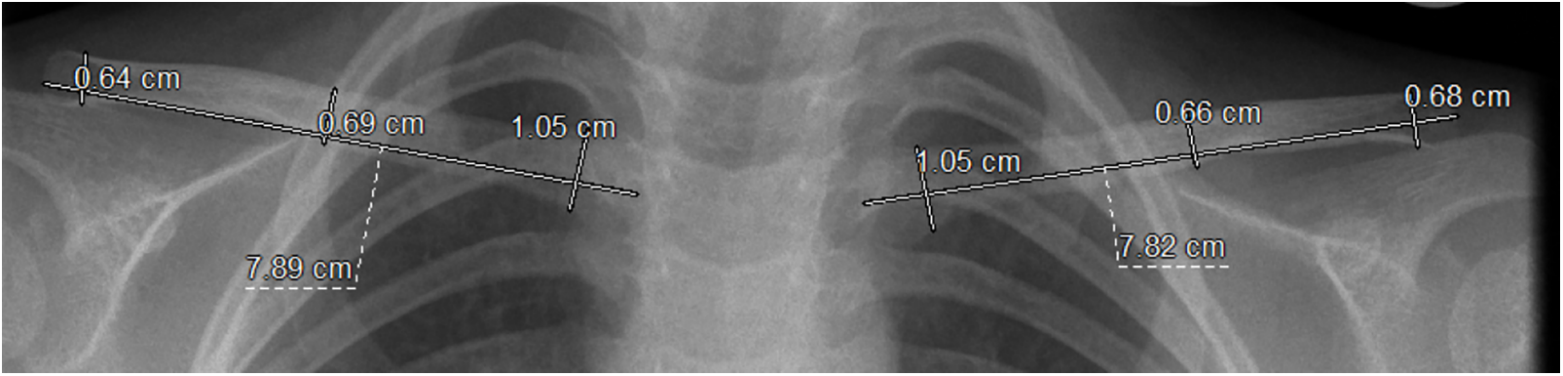
The process of measuring clavicle length and widths is illustrated on an anteroposterior chest x-ray using RIS/ PACS. Clavicle length is measured as the distance between the medial and lateral articular centers. Width measurements are taken 1 cm distal to the sternal end (medial width) and 0.5 cm proximal to the acromial end (lateral width). The central width is determined by dividing the distance between these two points in half.

**Figure 2:**
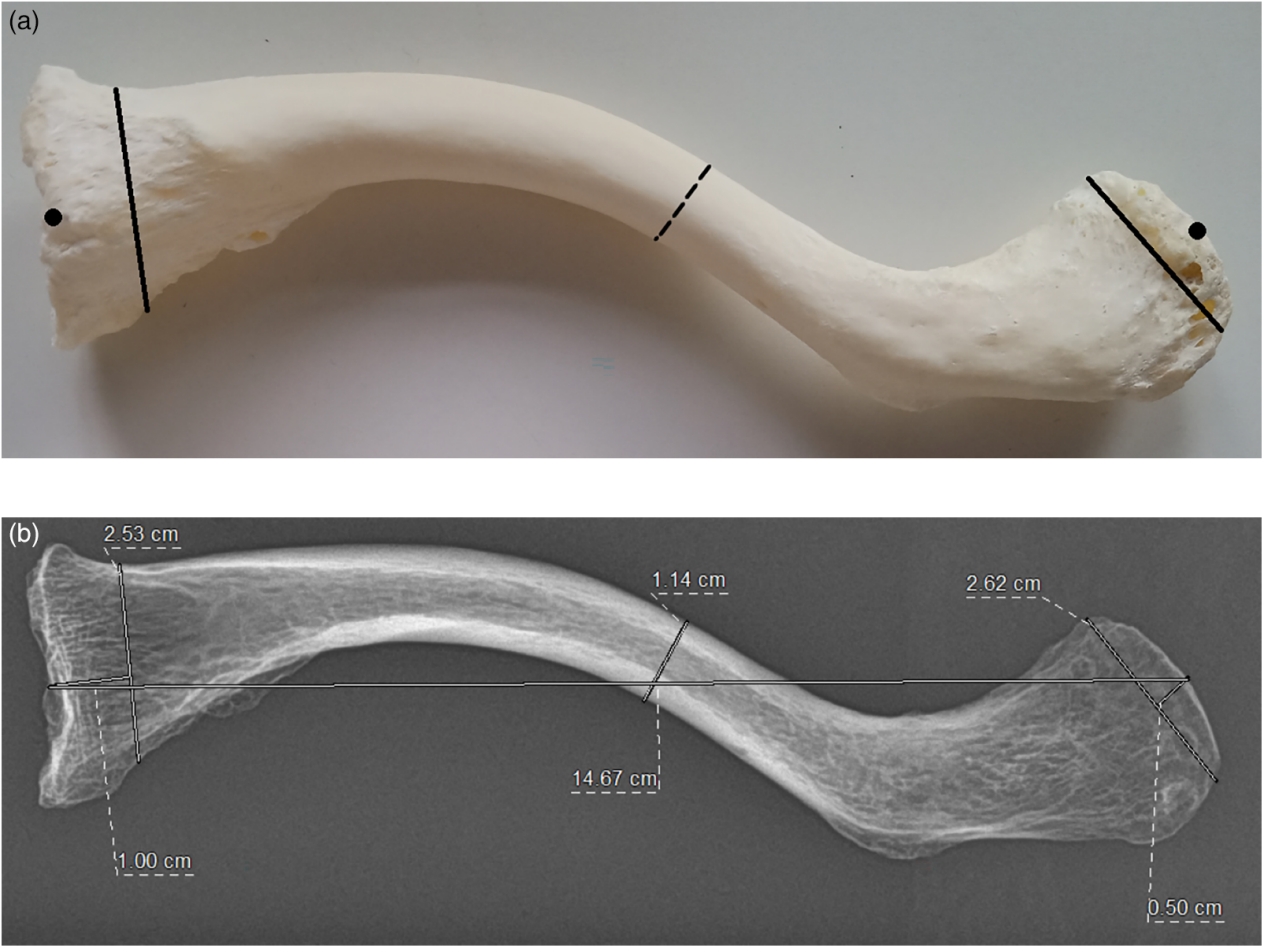
**(a)** The measurement protocol for manual assessment of “clavicle 1” was defined as follows: clavicular length was measured as the linear distance between the medial and lateral articular centers using precision calipers and a ruler (•). Medial clavicular width was assessed approximately 1 cm distal to the sternal end (−), while lateral clavicular width was measured 0.5 cm proximal to the acromial end (−). The central width was determined by identifying the midpoint between these two measurement points (…). **(b)** The measurement of the relevant anteroposterior x-ray images was performed using RIS/PACS. The macerated clavicles were assessed following the method described in **(a)**.

Displaying the x-ray images involved the use of a diagnostic monitor compliant with DIN6868-157 standards and the “Centricity™ Universal Viewer” (Centricity™ Universal Viewer Version 6.0) for clavicle measurements on all radiographs. Clavicle length was defined as the distance between the midpoints of the sternal and acromial articular surfaces. Width measurements were taken at specified distances, and central width was determined accordingly. Figure 1 provides an example of the measurement process using a chest x-ray. Figures 2a,b visually depict the procedures for both and physical and radiological measurements of a macerated clavicle.

Statistical analysis, conducted with SPSS Statistics (IBM SPSS Statistics 27.0), included *t*-tests to determine significances between age groups, sexes, and both sides of the body, with a significance level set at *P* < 0.05.

## 3 Results

A total of 1,340 clavicle pairs (right and left clavicle, respectively) were assessed for length and width based on chest radiographs, with radiographs categorized according to the sex assigned at birth among other factors. Measurements were conducted on 733 clavicle pairs from boys (54.7%) and 607 clavicle pairs from girls (45.3%).

To verify dimensional accuracy, clavicle measurements obtained using four radiographic techniques were compared with manual measurements. Table 2 outlines projection differences observed during this comparison.

**Table 2:** A comparative analysis of manual and radiological measurements of macerated clavicle bones is presented, highlighting the differences and correlations between the measurement techniques.

Examination type	Clavicle	Flat (0°)				Laterally lifted (−30°)			
		Width medial (in cm)	Width central (in cm)	Width lateral (in cm)	Length (in cm)	Width medial (in cm)	Width central (in cm)	Width lateral (in cm)	Length (in cm)
Manual measurement	1	2.6	1.2	2.9	14.3	–	–	–	–
	2	1.5	1.0	1.9	14.2	–	–	–	–
Mobile x-ray unit (in incubator); detector under clavicle	1	2.4	1.1	2.7	14.5	2.5	1.1	2.6	14.7
	2	1.6	1.0	1.9	14.8	–	–	–	–
Mobile x-ray unit (in incubator); detector in slide-in module	1	2.6	1.2	2.9	**16** **.** **0**	3.0	1.2	2.7	**16** **.** **0**
	2	1.8	1.0	1.8	**15** **.** **8**	–	–	–	–
Stationary x-ray unit; detector under clavicle; with grid	1	2.4	1.1	2.7	14.7	2.7	1.2	2.6	15.0
	2	1.7	1.0	2.1	14.3	–	–	–	–
Stationary x-ray unit; detector under clavicle; without grid	1	2.4	1.1	2.7	14.4	2.6	1.1	2.6	14.8
	2	1.6	1.0	2.0	14.1	–	–	–	–

The findings aim to evaluate the accuracy and reliability of each measurement approach in characterizing clavicular morphology.

The largest deviations between the manual measurements and X-ray techniques are emphasized in bold.

Significant differences in growth between the right and left clavicle were only evident in the “31st–34th week of gestation”, “5–8 months”, and “4–6 years” age groups, as detailed in Table 3. Notably, a larger length was observed for the left clavicle compared to the right in the “5–8 months” age group, whereas growth in the width of the central part was more pronounced on the right side in the other two age groups.

**Table 3:** Measurements of the left and right clavicles, highlighting significant discrepancies between the two are presented.

Measuring point	Age group	Mean ± SD (cm)		Mean difference in cm (left - right)	95% - confidence interval (difference)		*P*
					Lower value	Upper value	
Central width	31st–34th week of gestation	Left	0.30 ± 0.05	−0.01	−0.02	0.00	0.035
		Right	0.31 ± 0.05				
Length	5–8 month	Left	4.65 ± 0.60	0.11	0.04	0.18	0.004
		Right	4.55 ± 0.54				
Central width	4–6 years	Left	0.71 ± 0.01	−0.01	−0.02	0.00	0.002
		Right	0.72 ± 0.10				

Each entry includes the values for both clavicles and their difference. calculated as the left clavicle value minus the right. Gender distinctions are not considered.

**Figure 3:**
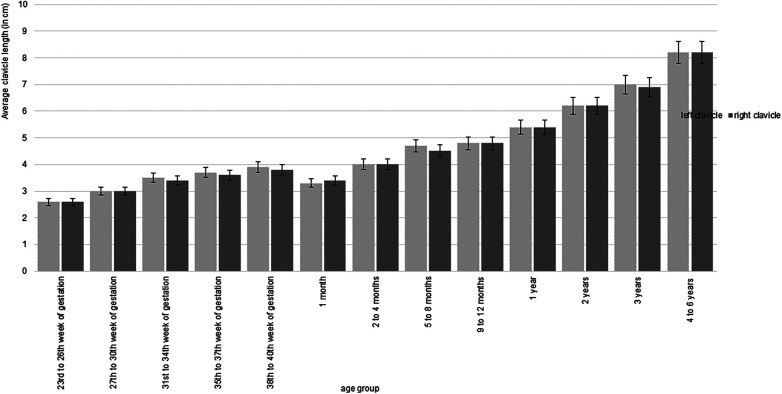
The average clavicle length, measured in centimeters on both sides of the body, is presented in relation to age groups, without distinguishing between sexes. A consistent increase in clavicle length is observed across age groups, except for the one-month age group. Overall, the lengths of the left and right clavicles are comparable and show no significant asymmetry.

A steady increase in clavicle length and width was observed from the “23rd–26th week of gestation” to “1 month.” However, a decrease in both parameters was noted in the one-month age group compared to preceding age groups. Subsequent age groups from “2–4 months” to “4–6 years” demonstrated continuous growth in clavicle length and width. Figure 3 illustrates the exact progression of clavicle length growth across the age groups, while clavicle width growth curves displayed an identical decrease in the one-month age group.

Significance testing between all age groups revealed unexpected findings attributed to decreasing values in the first month of life. Specifically, the “27th–30th week of gestation” age group exhibited significantly smaller clavicle lengths and widths compared to the “38th–40th week of gestation” group. However, no significant differences were observed compared to the subsequent “1 month” age group. Further analysis indicated that all age groups exhibited smaller clavicles compared to subsequent ones, with significance varying depending on the age groups compared.

**Figure 4:**
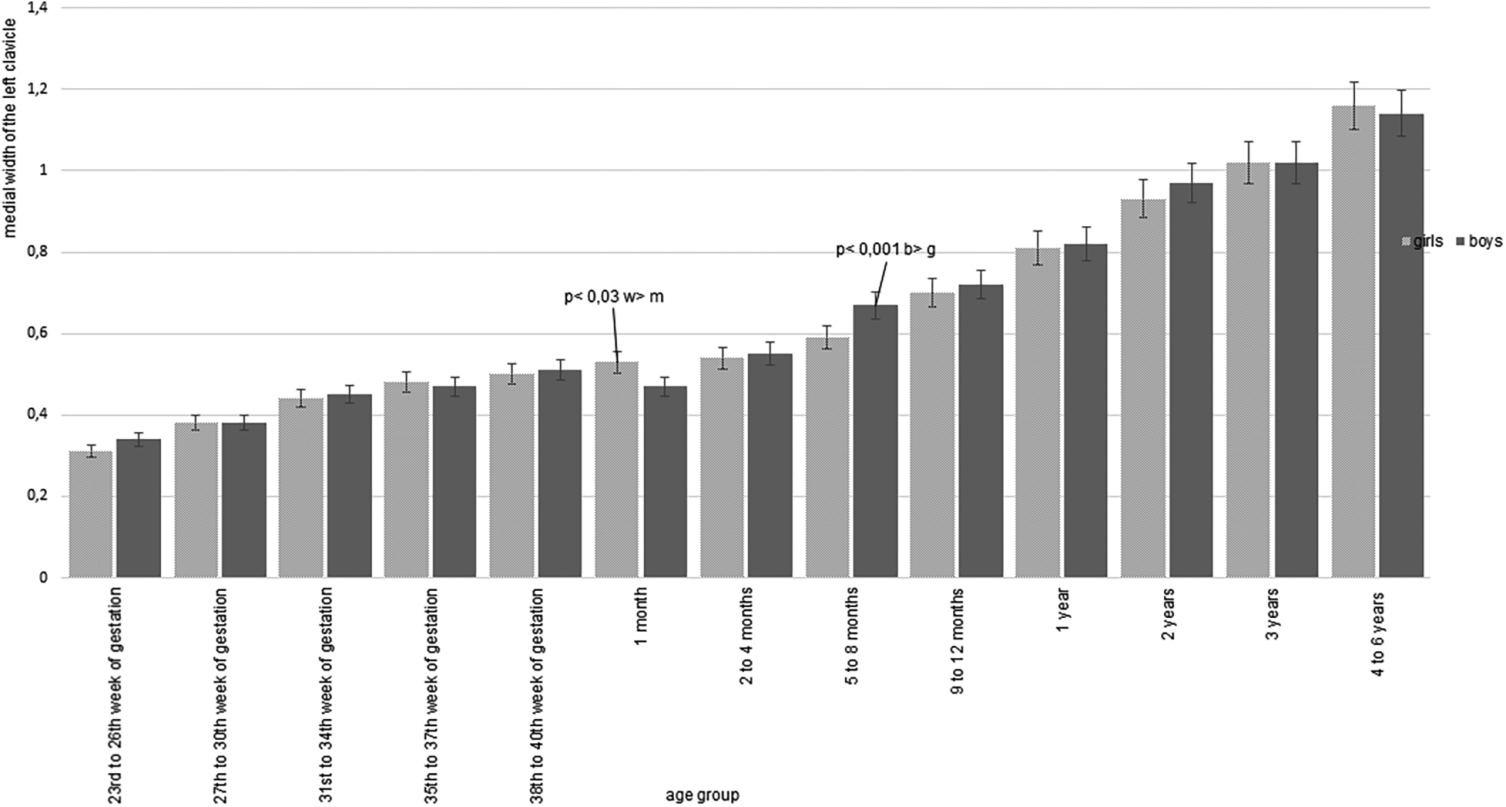
The medial width of the left clavicle, expressed in centimeters, is shown according to age group, with a separate analysis for both sexes. A consistent pattern of clavicle widening is evident across most age groups, apart from the one-month group. Notable sex-based differences are emphasized where statistically significant.

Additionally, measurements were stratified by the sex assigned at birth, revealing significant differences in clavicle widths across eight age groups, primarily in the second half of the first year of life. Boys' clavicles appeared wider than girls' in most instances, except at one month of age, where girls' clavicles appeared wider. Figure 4 shows the growth of the medial clavicle width on the left. Significances between both sexes are shown in Table 4.

**Table 4:** Statistically significant differences in the measured clavicular dimensions between boys and girls are shown.

Measuring point	Age group	Mean difference in cm (boys - girls)	95% - confidence interval (difference)		*P*
			Lower value	Upper value	
Medial width left	**1 month**	**−0** **.** **06**	**−0** **.** **11**	**−0** **.** **01**	**0** **.** **03**
	5–8 months	0.08	0.04	0.12	<0.001
Central width left	5–8 months	0.05	0.00	0.09	0.04
Lateral width left	4–6 years	0.03	0.00	0.06	0.04
Medial width right	5–8 months	0.08	0.03	0.13	<0.001
	2 years	0.04	0.00	0.06	0.03
Central width right	9–12 months	0.07	0.02	0.13	0.01
Lateral width right	9–12 months	0.05	0.01	0.10	0.02

Measurements were taken across all relevant age groups, with values reflecting both clavicle length and width. Significant variations are highlighted, illustrating gender-based discrepancies in clavicle development.

The instance where the clavicle size of girls exceeds that of boys during the first month is highlighted in bold.

## 4 Discussion

Measurements conducted on macerated clavicle bones using four common radiographic techniques displayed no consistent differences compared to manual measurements, regardless of the detector position. This suggests that measurements based on x-ray images yield realistic values.

As anticipated, clavicle growth generally exhibited a steady increase in length and width across the studied age groups, with deviations observed in one-month-old patients, particularly in males.

Preterm infants were categorized as one-month-olds from 29 days of age, a classification that mirrored full-term infants, although the actual age attainment occurred after a corrective period. Consequently, their clavicles, due to slower growth or smaller initial size, did not demonstrate age-appropriate development compared to fully mature one-month-old infants at the time of evaluation. This limitation applied to all age groups; however, the increasing convergence of growth trajectories between premature and full-term infants rendered this distinction less significant for our analyses (8, 9).

Significant differences in the growth of left and right clavicles were identified in three age groups, potentially indicative of stimulus-induced, laterally uneven growth or the manifestation of handedness (10–15).

Inconsistent findings regarding asymmetrical clavicle growth have been reported in the literature. While Black and Scheuer observed a slight length discrepancy, with the left clavicle being longer than the right, McGraw et al. and Aira et al. presented conflicting results (16–18). Conversely, Ogata and Uhthoff as well as Wisniewski et al. found no evidence of lateral differences in their respective studies (19, 20). Given that significant side-dependent growth was only evident in three cases in our study, mean values of clavicle lengths and widths were utilized for simplification and further analysis.

Previous studies have reported varying results on gender-specific clavicle growth.

Significant differences in clavicle dimensions between boys and girls have been consistently reported in studies by Aira, McGraw, Qui, and Yang et al. (16, 18, 21, 22). However, other studies have found no significant disparities in clavicle growth between the sexes (20, 23).

Our findings align with those studies that suggest boys generally have slightly wider and longer clavicles than girls (24–26), though these differences were minimal and not statistically significant in most age groups. Notably, an exception was observed in one-month-old infants, where chest radiographs consistently showed larger clavicle dimensions in girls, with a significant difference noted only in the medial width of the left clavicle.

These findings can be interpreted through the lens of preterm birth distribution. Research by Rosa et al. indicates a higher incidence of preterm births among male fetuses in the United States, a trend corroborated by our study's observation of a higher number of preterm-born boys compared to girls (27). Additionally, Glass et al. noted that general developmental outcomes following preterm birth are more favorable in girls than in boys, potentially accounting for the better-developed clavicles observed in girls within our study cohort (28).

The observation that boys' clavicles are generally wider and longer than those of girls, irrespective of the aforementioned exception, becomes more pronounced in our study from the latter half of the first year of life. This trend is supported by WHO growth charts, which indicate that the disparity in overall body size between genders typically emerges around four months of age and persists throughout subsequent months (25, 29). Black and Scheuer's study further reinforces this notion, demonstrating a notably accelerated growth in clavicle length from the sixth month of life until the completion of the first year, compared to the initial six months (17). This growth is also more pronounced compared to subsequent age groups. Although previous studies did not explicitly focus on sex-assigned at birth or clavicle width measurements, our findings suggest that clavicle width growth is also influenced by this growth spurt.

We verified our measured values by directly comparing them with those obtained from other studies. These comparison values included measurements obtained through manual measurements using anatomical skeleton collections (17, 30, 31), measurements from two- and three-dimensional radiographs (18, 20), and measurements from sonographic images (32, 33).

All comparative studies described continuously increasing growth curves (17, 20, 30–33). However, the exact measured values and the number of clavicles measured differed considerably between the studies. Generally, our measurements fell within the range of values reported by other studies, confirming their accuracy. Exceptions were noted when comparing clavicle widths with those reported by Corrigan, Mohsin et al., and Wisniewski et al. (20, 30, 31). In these cases, deviations of more than 2 mm in both directions were observed. Since clavicle width was not a primary focus in the other studies, a precise assessment to validate these values was not possible.

While studies relying on manual measurements are typically deemed the most accurate due to the ability to view the bone in its true form, our findings demonstrate that x-ray images also offer a realistic depiction of the clavicle bone. Consequently, our measured values can be considered sufficiently realistic, serving as a means to validate values reported in other studies. Moreover, our study's extensive measurement of clavicles across various age groups allows for the establishment of reliable average values, thereby serving as a valuable reference for future investigations.

These measured values not only illustrate the typical development of clavicle lengths and widths but also have the potential to expedite the detection of dysplasias. Furthermore, they can serve as reference points for age determination in forensic contexts, facilitating more accurate assessments.

However, our study has some limitations. The small number of clavicle measurements available from other studies only permits validation within a general range, making precise statements about exact values difficult. Furthermore, clavicle widths were often not considered, or the specific measurement methods were not clearly defined, which restricts the scope of comparisons. Measuring radiographs, particularly in neonates and infants, was challenging due to the limited availability of suitable chest x-rays. Consistent with previous studies, measurements were defined as linear distances between two points, thus overlooking the individual curvature of the clavicle in each dimension.

## 5 Conclusion

The anticipated, consistently rising developmental trajectories of the clavicle were validated, except for the initial month of life. Additionally, notable discrepancies were observed between body sides and genders. Given that clavicle measurements derived from radiographs yield realistic values, establishing reference standards for clavicle growth in premature infants, newborns, as well as infants and toddlers becomes feasible. These reference values can prove instrumental in diagnosing deformities or facilitating forensic age estimation. Despite the limitations mentions, identifiable trends emerged that warrant further investigation. Future studies should also consider age correction for preterm infants.

## Data Availability

The raw data supporting the conclusions of this article will be made available by the authors, without undue reservation.
